# Identifying classifier input signals to predict a cross-slope during transtibial amputee walking

**DOI:** 10.1371/journal.pone.0192950

**Published:** 2018-02-16

**Authors:** Courtney E. Shell, Glenn K. Klute, Richard R. Neptune

**Affiliations:** 1 Department of Mechanical Engineering, The University of Texas at Austin, Austin, Texas, United States of America; 2 Department of Veterans Affairs, Puget Sound Health Care System, Seattle, Washington, United States of America; 3 Department of Mechanical Engineering, University of Washington, Seattle, Washington, United States of America; Northwestern University, UNITED STATES

## Abstract

Advanced prosthetic foot designs often incorporate mechanisms that adapt to terrain changes in real-time to improve mobility. Early identification of terrain (e.g., cross-slopes) is critical to appropriate adaptation. This study suggests that a simple classifier based on linear discriminant analysis can accurately predict a cross-slope encountered (0°, -15°, 15°) using measurements from the residual limb, primarily from the prosthesis itself. The classifier was trained and tested offline using motion capture and in-pylon sensor data collected during walking trials in mid-swing and early stance. Residual limb kinematics, especially measurements from the foot, shank and ankle, successfully predicted the cross-slope terrain with high accuracy (99%). Although accuracy decreased when predictions were made for test data instead of the training data, the accuracy was still relatively high for one input signal set (>89%) and moderate for three others (>71%). This suggests that classifiers can be designed and generalized to be effective for new conditions and/or subjects. While measurements of shank acceleration and angular velocity from only in-pylon sensors were insufficient to accurately predict the cross-slope terrain, the addition of foot and ankle kinematics from motion capture data allowed accurate terrain prediction. Inversion angular velocity and foot vertical velocity were particularly useful. As in-pylon sensor data and shank kinematics from motion capture appeared interchangeable, combining foot and ankle kinematics from prosthesis-mounted sensors with shank kinematics from in-pylon sensors may provide enough information to accurately predict the terrain.

## Introduction

Uneven terrain environments are commonly encountered in activities of daily living. Cross-slopes are one such terrain prevalent while walking in both man-made and natural environments. Despite accessibility standards requiring that cross-slopes not exceed 5% [1], surveys have shown that approximately 25% of sidewalks in US cities do not meet those standards [2]. Cross-slopes are also prevalent in natural terrain, and backcountry trails often have cross-slopes up to 70% [3]. When walking on a cross-slope, healthy subjects adapt to terrain changes by modulating their ankle joint angles and moments to conform their foot to the slope, maximize their base of support and control their center of pressure (COP) trajectory [4, 5].

Lower-limb amputees rely on their prosthetic foot to restore mobility, and thus it is important that the prosthetic foot provide the functions of the anatomical ankle-foot to allow natural navigation in challenging environments. Most commercially-available prosthetic feet have a passive stiffness profile that does not adjust foot-floor interactions in response to uneven or unexpected terrain. However, recent research has focused on developing semi-active or active prosthetic feet capable of altering their dynamic response based on terrain. Semi-active feet adjust prosthesis properties during gait, such as the neutral angle (e.g., [6, 7]) or stiffness (e.g., [8]), while active feet have electric or hydraulic actuators that control the ankle joint during gait (e.g., [9–11]). Pairing these devices with feedback control and/or terrain prediction algorithms would create a prosthesis that better emulates the anatomical ankle-foot.

Various control algorithms have been developed for semi-active and active prostheses. Task-specific controllers have been explored for locomotion, (i.e., level-walking, stair-climbing, incline and decline walking) as well as sitting and standing, with coordination and switching between gait-phase-specific controllers often performed by finite state machines (for review, see [12]). Previous studies have successfully integrated pattern-recognition algorithms into control schemes to determine locomotion mode (e.g., walking on level-ground, stairs or slopes [13–15]). However, methods of identifying a cross-slope so that the dynamic response of the prosthetic foot can be adapted to the side-to-side terrain variation have been largely unexplored.

Efforts to create accurate locomotion mode prediction algorithms have included exploration of different pattern-recognition algorithms and input signal selection. In many cases, linear discriminant analysis (LDA) provides comparable accuracy with less computational expense than other techniques [16–18]. Various input signal combinations have been explored including myoelectric, mechanical, ground reaction force, and computer vision (e.g., [13, 14, 16, 19–21]. While algorithms that use a combination of myoelectric and mechanical signals have been shown to provide more accurate locomotion mode detection and transition between modes than myoelectric or mechanical signals alone [13–15], measuring in-socket myoelectric signals presents added complications, including patient comfort, motion artifact, and challenges for long-term application outside of a laboratory environment [15, 22]. In contrast, semi-active and active devices are already equipped with mechanical sensors used for actuator control and additional position encoders and inertial measurement devices would be relatively simple to add with minimal impact on foot performance or patient comfort.

The goal of this study was to determine kinematic and kinetic signals that could be used by an LDA classifier to accurately identify the cross-slope terrain encountered without the aid of myoelectric signals. To compare the usefulness of different input signals, classifiers were developed using various combinations of data including only motion capture data, only measurements from sensors embedded in the pylon, and both motion capture data and in-pylon sensor measurements. Individual input signals were ranked based on the effectiveness of the signal to improve classifier accuracy. Greater classification accuracy was expected when classifiers were evaluated using a randomly-excluded portion of the training data rather than a separate set of test data. However, input signals selected based on classifier accuracy evaluated using the training data were not expected to vary greatly from those selected based on evaluation with a separate set of test data. Since in-pylon sensor and motion capture data were anticipated to be of comparable quality, similar input signal selection was expected using the portable in-pylon sensor data instead of analogous motion capture data.

## Materials and methods

LDA classifiers were trained on previously-collected experimental data measuring the biomechanical response of amputees to a step on cross-sloped terrain with their clinically prescribed ankle-foot prosthesis. Since classification accuracy can degrade when a new subject uses a pattern recognition algorithm trained on a different set of subject data [23], input signal selection choice was compared using classifier accuracy evaluated with a randomly-excluded portion of the training data (i.e., leave-one-out cross-validation, LOOCV) and also evaluated with two test datasets. The Training Data were collected from subjects walking with their clinically prescribed ankle-foot prosthesis when they could see the cross-slope. Test Set 1 was collected from a subject walking with his clinically prescribed ankle-foot prosthesis when he could not see the cross-slope. Since the classifier will eventually be paired with a new prototype ankle-foot prosthesis that uses its prediction to adapt to terrain encountered [24], Test Set 2 was collected while two subjects walked with the new prototype prosthesis with adaptation disabled when they could see the cross-slopes. Classifiers trained using data from only in-pylon sensors were compared with those trained using motion capture data to investigate the effects of using portable sensor data as a step toward implementation outside the laboratory.

### Experimental data collection

Experimental data were previously collected at the Department of Veterans Affairs Center for Limb Loss and Mobility in a protocol approved by the Department of Veterans Affairs internal review board. After obtaining written documentation of informed consent, three male, traumatic unilateral transtibial amputees (50±15 years old, 83.3±7.7 kg, 1.80±0.03 m, 27±19 years since amputation, 2 left/1 right) walked with their clinically prescribed, passive energy-storage-and-return prosthesis across five force plates (Advanced Mechanical Technology, Inc., Watertown, MA) on a 10-m walkway where the middle force plate was rotated to provide a 0°, +15° or -15° cross-slope [25]. When set at +15° the cross-slope caused ankle inversion and when set at -15° it caused ankle eversion. Data were collected from all three subjects when they could see the middle force plate configuration (S3 Dataset: Training Data). Data were also collected from one of the subjects when he was blinded to the middle force plate configuration by obscuring it from view with an opaque, elastic material (S1 Dataset: Test Set 1). The three cross-slope configurations were tested in a random order with a single acclimation trial then 4 to 6 trials in a block, where subjects took one step on the cross-slope in a trial. Three-dimensional (3D) marker and ground reaction force data were recorded at 120 Hz and 1200 Hz, respectively, using a 12-camera motion capture system (Vicon Nexus, Vicon Motion Systems, Oxford, UK). In addition to the Vicon Plug-in-Gait marker set, markers were placed bilaterally on the medial knee epicondyle, medial malleolus, tibial tuberosity, fibular head, and first and fifth metatarsal heads. Clusters of four markers were also placed bilaterally on the upper arms and thighs. Trials with at least five knee flexion-extension movements were collected to find the knee functional joint axes [26] (Visual 3D, C-Motion, Inc., Germantown, MD). Marker and GRF data were filtered using a 4^th^-order, low-pass Butterworth filter with cutoff frequencies of 6 and 20 Hz, respectively.

Joint angles, moments and powers as well as shank and foot segment velocities, angular velocities and accelerations were calculated in Visual 3D using standard inverse dynamics with a model scaled to the subject’s height and mass. Data were time-normalized to the gait cycle. GRFs and joint moments were also normalized to the subject’s body weight. Residual ankle joint angles were normalized to the ankle angles during swing when the prosthesis was in its unloaded configuration to minimize bias due to variations in alignment. Ankle angular velocity and acceleration were calculated as the first and second time derivatives of the ankle angle. Shank acceleration and angular velocity were also collected at 120 Hz from a sensor package custom-built at the Department of Veterans Affairs Center for Limb Loss and Mobility. The sensor package was located inside the pylon and included a tri-axial accelerometer and bi-axial rate gyroscope along with batteries, a microcontroller, and an SD memory card [27]. Raw data from the accelerometer and gyroscope were used. Acceleration and angular velocity collected by the in-pylon sensors were with respect to a shank reference frame while those calculated with Visual3D were with respect to the fixed laboratory reference frame.

Two subjects also completed the protocol wearing a prototype ankle-foot prosthesis with variable coronal-plane stiffness [24] when they could see the middle force plate configuration (S2 Dataset: Test Set 2). The three cross-slope configurations were tested in a random order with a single acclimation trial then 4 to 5 trials in a block, where subjects took one step on the cross-slope in a trial. For this study, the prosthesis coronal-plane stiffness was set to a single stiffness level. Thus, the prototype ankle-foot prosthesis acted much like the subjects’ clinically-prescribed energy-storage-and-return prosthetic foot.

### Classifier design

**Fig 1:**
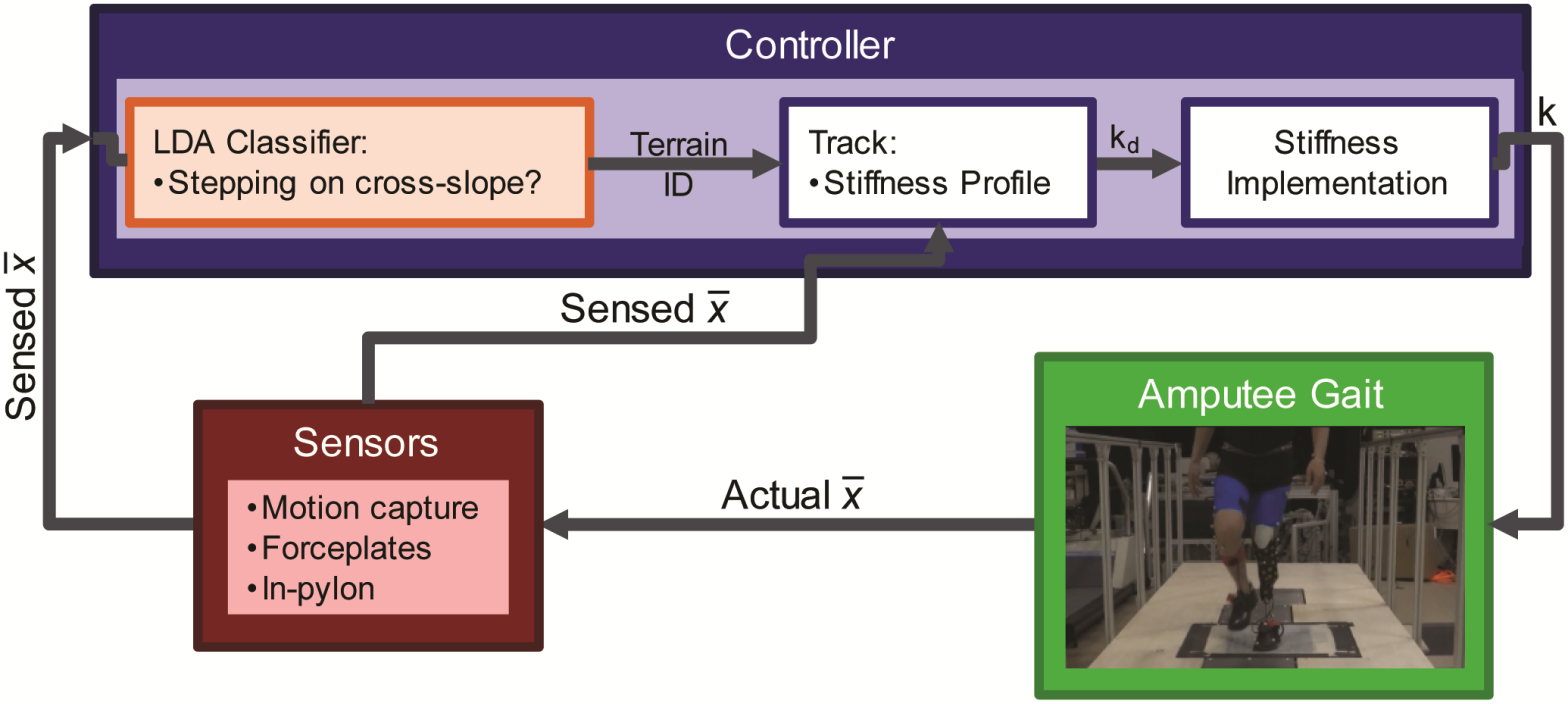
Control scheme diagram. The LDA classifier will identify the cross-slope encountered using measured kinematics and kinetics ($\bar{x}$). A mid-level controller will use this information to provide a desired stiffness (k_d_) to the lower-level controller that will modify the stiffness profile (k) of the prototype variable-stiffness prosthetic foot.

An LDA classifier was designed to determine if the subject was stepping on a cross-slope or level-ground as one component of the control scheme for a semi-active prosthesis (Fig 1). Classifiers with various combinations of input signals were trained and evaluated using LOOCV (MATLAB, The MathWorks, Inc., Natick, MA) with measurements from three subjects. Performance was also evaluated using two test datasets with slight modifications to the experimental data collection setup to explore how input signal selection and classifier predictive ability might change in these different conditions. Test Set 1, where the subject could not see the cross-slope, explored the effects of when the cross-slope is unexpected and possible anticipatory modifications are therefore absent. Test Set 2, where subjects could see the cross-slopes and walked with a prototype semi-active prosthesis set to a single stiffness level, was included to explore the effects of the prototype prosthetic foot, which even with adaptation disabled, has a slightly different underlying stiffness profile and ankle center of rotation than the prescribed prostheses subjects used in the Training Data.

**Fig 2:**
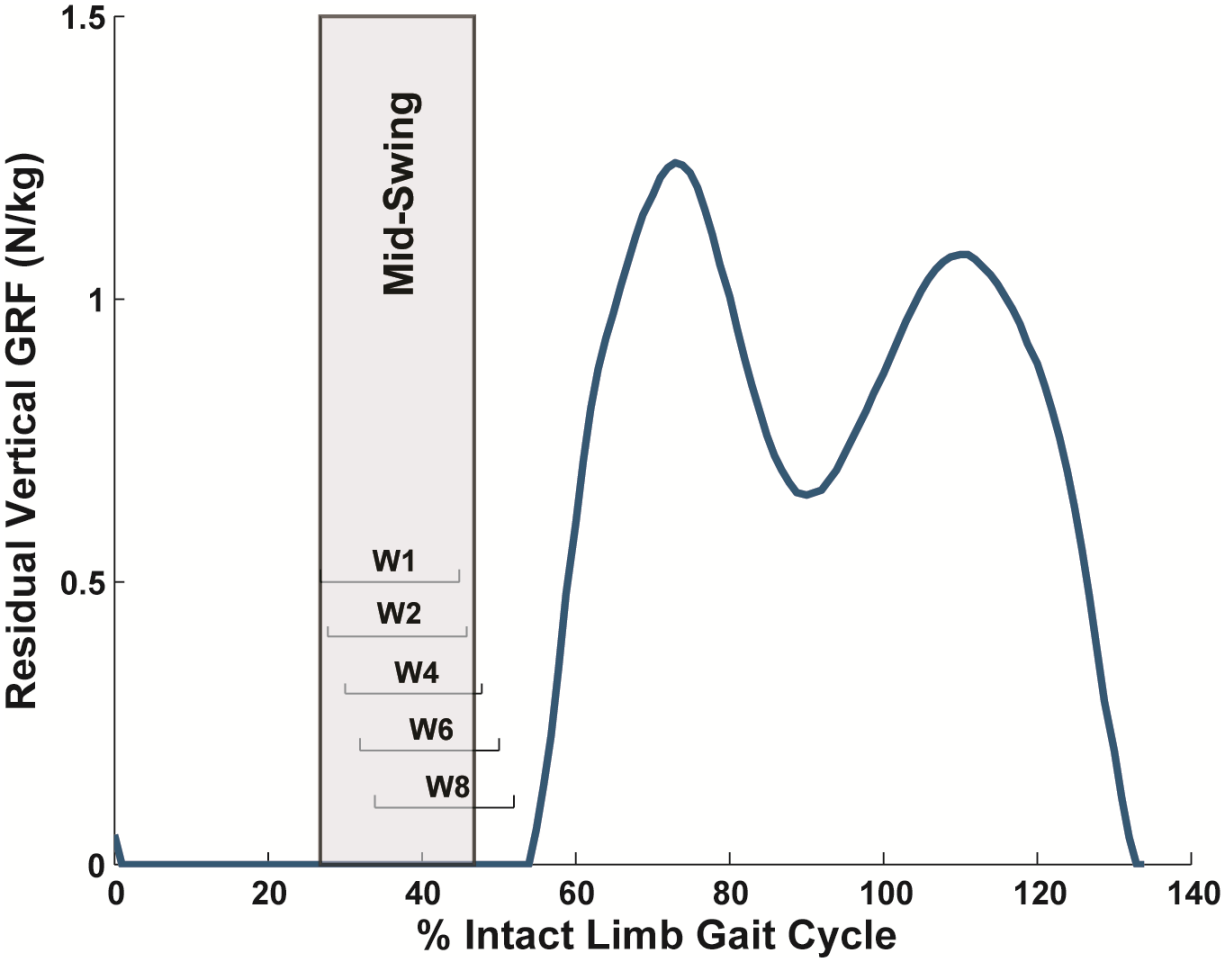
Overlapping 150-ms windows of data (W1, W2, etc.) were categorized into regions. Mid-swing windows had at least half of the data collected during mid-swing and less than half of the data collected after residual limb heel-strike.

Various combinations of input signals from the residual limb in mid-swing, including kinematics and kinetics from both motion capture and in-pylon sensors, were assessed to determine which signals provided the most accurate prediction of the cross-slope terrain (S1 Table). Data were exported from Visual3D at the original sampling rate and examined in windows of 150 ms that overlapped and were incremented forward every 8.33 ms. In each window, mean, standard deviation, maximum and minimum values were calculated for joint kinematics, joint moments, joint powers and segment kinematics (MATLAB, The MathWorks, Inc., Natick, MA). Mean and standard deviation values were also calculated for ground reaction force and center of pressure data. These signal characteristics are conducive to real-time calculations and have been useful in previous locomotion-mode classifiers [13, 20] and intent recognition [14, 15]. Values from the various signals were combined into a single vector for the window, which consisted of 124 values in the full dataset and 24 values in the in-pylon-sensor-only dataset, and then examined by the classifier. Windows of data were categorized as mid-swing and used for classification if at least half the data were collected during mid-swing (windows beginning 192 to 250 ms before heel strike) and less than half of the data were collected after residual limb heel-strike (windows ending 75 ms after heel strike) (Fig 2).

Since the classifier can leverage information provided by combinations of input signals in unforeseen ways, input signals selected using two different selection methods (sequential forward selection (SFS) and sequential backward selection (SBS)) were compared [14]. Classifier performance was evaluated with confusion matrices. Input signals were ranked by their effect on overall classifier accuracy, defined as the number of observations for which terrain was predicted correctly divided by the total number of observations in the dataset. SFS evaluated classifiers based on each signal alone then selected the signal that produced the most accurate mid-swing classifier. This process was repeated by adding each of the remaining signals, training and evaluating classifiers to determine which signal produced the most accurate classifier when combined with those already chosen, and selecting that signal until all input signals were added. SBS started by using all but one of the signals in the classifiers evaluated, sequentially removing each of the signals and choosing to remove the one that led to the least accurate classifier. This process was repeated until only one signal was used in the classifier evaluated. Thus, two different rankings of input signal importance to classifier accuracy were obtained.

## Results

### Input signal rankings

Input signals had different contributions to classifier accuracy when selected using SBS instead of SFS and when classifiers were evaluated using different datasets (Tables 1–3). Rankings of the input signals were generally more similar between SBS and SFS when a given dataset was used for evaluation than between different evaluation datasets, although the rankings were more varied when a larger number of potential input signals were analyzed (Tables 1 and 2). When evaluated using LOOCV, the rank of the input signals required to reach 95% accuracy was the same when in-pylon sensor data was substituted with the shank acceleration and angular velocity calculated from motion capture data (Table 3). The number of additional signals required to reach 99% accuracy were similar with and without the in-pylon sensor data for SBS, although the additional signals were not the same.

**Table 1:** Rankings of input signals found using sequential forward selection (SFS) and sequential backward selection (SBS) when only in-pylon sensor data were used. The two algorithms selected signals in the same order except when Test Set 2 was used to evaluate classifier accuracy.

		Input Signal		Error	
		SFS	SBS	SFS	SBS
**LOOCV**	1	ML Acc		0.47	
	2	Cor AngVel		0.37	
	3	InfSup Acc		0.32	
	4	AP Acc		0.26	
	5	Sag AngVel		0.24	
	6	Tran AngVel		0.22	
**Test Set 1**	1	InfSup Acc		0.51	
	2	AP Acc		0.44	
	3	Sag AngVel		0.36	
	4	Tran AngVel		0.42	
	5	ML Acc		0.49	
	6	Cor AngVel		0.64	
**Test Set 2**	1	Cor AngVel		0.47	
	2	ML Acc		0.46	
	3	InfSup Acc	AP Acc	0.47	
	4	AP Acc	Tran AngVel	0.47	0.44
	5	Sag AngVel		0.49	0.45
	6	Tran AngVel	InfSup Acc	0.52	

LOOCV, classifiers were evaluated using leave-one-out cross-validation with the training data from three subjects walking with their clinically prescribed ankle-foot prosthesis when they could see the configuration of the cross-slope; Test Set 1, classifiers were evaluated using data from a subject walking with his clinically prescribed ankle-foot prosthesis when he could not see the configuration of the cross-slope; Test Set 2, classifiers were evaluated using data from two subjects walking with the prototype ankle-foot prosthesis when they could see the configuration of the cross-slope; Definitions: AngVel, residual limb shank angular velocity; Acc, residual limb shank acceleration; Cor, coronal plane; Tran, transverse plane; Sag, sagittal plane; AP, anteroposterior direction; InfSup, inferior-superior direction; ML, mediolateral direction.

**Table 2:** Rankings of input signals found using sequential forward selection (SFS) and sequential backward selection (SBS) when classifiers were trained on the expanded set of input signals from three subjects walking with their clinically prescribed ankle-foot prosthesis when they could see the configuration of the cross-slope and evaluated using data from two different test sets.

	SFS		SBS	
	Test Set 1	Test Set 2	Test Set 1	Test Set 2
1	Ankle Inversion ω	Foot Vert Velocity	Ankle Inversion α	Shank Vert Velocity
2	Ankle Flexion α	Vert GRF	Ankle Flexion α	Foot ML AngVel
3	Ankle Inversion α	ML GRF	Foot AP Velocity	Foot Vert Acc
4	Foot Vert AngVel	IPS Cor AngVel	Shank AP Velocity	Ankle Inversion
5	Ankle Inversion Power	Ankle Inversion Power	Shank Vert Velocity	Foot ML Acc
6	Vert COP	Shank AP Velocity	IPS InfSup Acc	IPS Cor AngVel
7	Vert GRF	Foot Vert Acc	Ankle Inversion ω	ML COP
8	ML GRF	IPS Tran AngVel	IPS Cor AngVel	IPS AP Acc
9	Ankle Flexion Power	Foot ML Acc	Ankle Flexion	Foot AP AngVel
10	AP GRF	Ankle Inversion ω	Foot ML Acc	IPS ML Acc
11	AP COP	IPS Sag AngVel	Foot Vert AngVel	Foot Vert Velocity
12	Shank ML Velocity	Foot ML AngVel	Foot Vert Velocity	Foot AP Velocity
	Shading Legend			
	Signal among top 12:	Both selection methods, both test sets for one method	Both selection methods, one test set	Both test sets, one selection method

Test Set 1, classifiers evaluated using data from a subject walking with his clinically prescribed ankle-foot prosthesis when he could not see the configuration of the cross-slope; Test Set 2, classifiers evaluated using data from two subjects walking with the prototype ankle-foot prosthesis when they could see the configuration of the cross-slope; Definitions: ω, angular velocity; α, angular acceleration; GRF, ground reaction force; COP, center of pressure; ML, mediolateral direction; AP, anteroposterior direction; Vert, vertical direction; InfSup, inferior-superior direction; AngVel, angular velocity; Acc, acceleration; IPS, measurement made by in-pylon sensors; Cor, coronal plane; Tran, transverse plane; Sag, sagittal plane. Input signals above the single solid lines are required for classifier accuracy to be greater than 95%.

**Table 3:** Rankings of the top ten input signals found using sequential forward selection (SFS) and sequential backward selection (SBS) when in-pylon sensor data was (IPS) and was not (MC) included for classifiers trained on the full set of trial input signals from three subjects walking with their clinically prescribed ankle-foot prosthesis when they could see the configuration of the cross-slope and evaluated using leave-one-out cross-validation (LOOCV).

	SFS		SBS	
	In-Pylon Sensor + Motion Capture Data	Only Motion Capture Data	In-Pylon Sensor + Motion Capture Data	Only Motion Capture Data
1	Ankle Inversion α	Ankle Inversion α	Ankle Inversion ω	Ankle Inversion ω
2	Shank Vert Velocity	Shank Vert Velocity	Foot Vert Acc	Foot Vert Acc
3	Ankle Flexion	Ankle Flexion	Foot ML AngVel	Foot ML AngVel
4	IPS AP Acc	Ankle Flexion α	Foot AP AngVel	Foot AP AngVel
5	Foot ML Velocity	Shank Vert AngVel*	Shank AP Velocity	Shank AP Velocity
6	Foot Vert Velocity	Shank Vert Acc*	Ankle Flexion	Foot ML Acc
7	Ankle Inversion	Ankle Inversion ω	ML COP	Shank Vert AngVel*
8	IPS Tran AngVel	Shank ML Velocity	Foot Vert AngVel	ML COP
9	Ankle Inversion ω	Shank ML Acc*	Foot ML Acc	AP COP
10	Ankle Inversion Moment	Foot Vert Acc	Foot AP Velocity	Foot AP Velocity
	Shading Legend			
	Signal among top 12:	Both datasets, both selection methods	Both datasets, both selection methods for one dataset	Both datasets, one selection method

Definitions: ω, angular velocity; α, angular acceleration; COP, center of pressure; ML, mediolateral direction; AP, anteroposterior direction; Vert, vertical direction; AngVel, angular velocity; Acc, acceleration; Tran, transverse plane. Single and double solid lines indicate the input signals required for the classifier accuracy to be greater than 95% and 99%, respectively.

* indicates an input signal calculated using motion capture data that was substituted for an analogous signal measured using in-pylon sensors.

### Classifier accuracy

Using only in-pylon sensor data, classifier accuracy when evaluated using LOOCV ranged from 63% to 78% and prediction accuracy for individual terrains was between 59% and 87% (Table 4 and S2 Table). Classifier and prediction accuracy using IPS and evaluated using LOOCV generally increased as more signals were added. Accuracy degraded when evaluated using the test datasets (Table 4).

**Table 4:** Overall accuracy (All) and range of accuracy for individual cross-slope terrain (Ind.) for classifiers with 2, 3, 4, 5 and 6 input signals that exhibited the highest overall accuracy identifying the cross-slope terrain when only information from the in-pylon sensors was used.

Input Signals			Accuracy		
			LOOCV Mid-Swing	Test Set 1 Mid-Swing	Test Set 2 Mid-Swing
IPS ML Acc	IPS Cor AngVel	All	0.63	0.33	0.54
		Ind.	0.59–0.67	0.06–0.49	0.28–0.73
IPS InfSup Acc IPS ML Acc	IPS Cor AngVel	All	0.68	0.37	0.53
		Ind.	0.67–0.71	0.23–0.54	0.30–0.88
IPS AP Acc IPS InfSup Acc IPS ML Acc	IPS Cor AngVel	All	0.74	0.36	0.53
		Ind.	0.69–0.79	0.21–0.43	0.12–0.74
IPS AP Acc IPS InfSup Acc IPS ML Acc	IPS Cor AngVel IPS Sag AngVel	All	0.76	0.43	0.51
		Ind.	0.73–0.81	0.30–0.56	0.20–0.77
IPS AP Acc IPS InfSup Acc IPS ML Acc	IPS Cor AngVel IPS Tran AngVel IPS Sag AngVel	All	0.78	0.36	0.48
		Ind.	0.71–0.87	0.26–0.52	0.36–0.61

LOOCV, classifier accuracy evaluated using leave-one-out cross-validation with the training data from three subjects walking with their clinically prescribed ankle-foot prosthesis when they could see the configuration of the cross-slope; Test Set 1, classifier accuracy evaluated using data from a subject walking with his clinically prescribed ankle-foot prosthesis when he could not see the configuration of the cross-slope; Test Set 2, classifier accuracy evaluated using data from two subjects walking with the prototype ankle-foot prosthesis when they could see the configuration of the cross-slope; Definitions: AngVel, residual limb shank angular velocity; Acc, residual limb shank acceleration; Cor, coronal plane; Tran, transverse plane; Sag, sagittal plane; AP, anteroposterior direction; InfSup, inferior-superior direction; ML, mediolateral direction.

**Fig 3:**
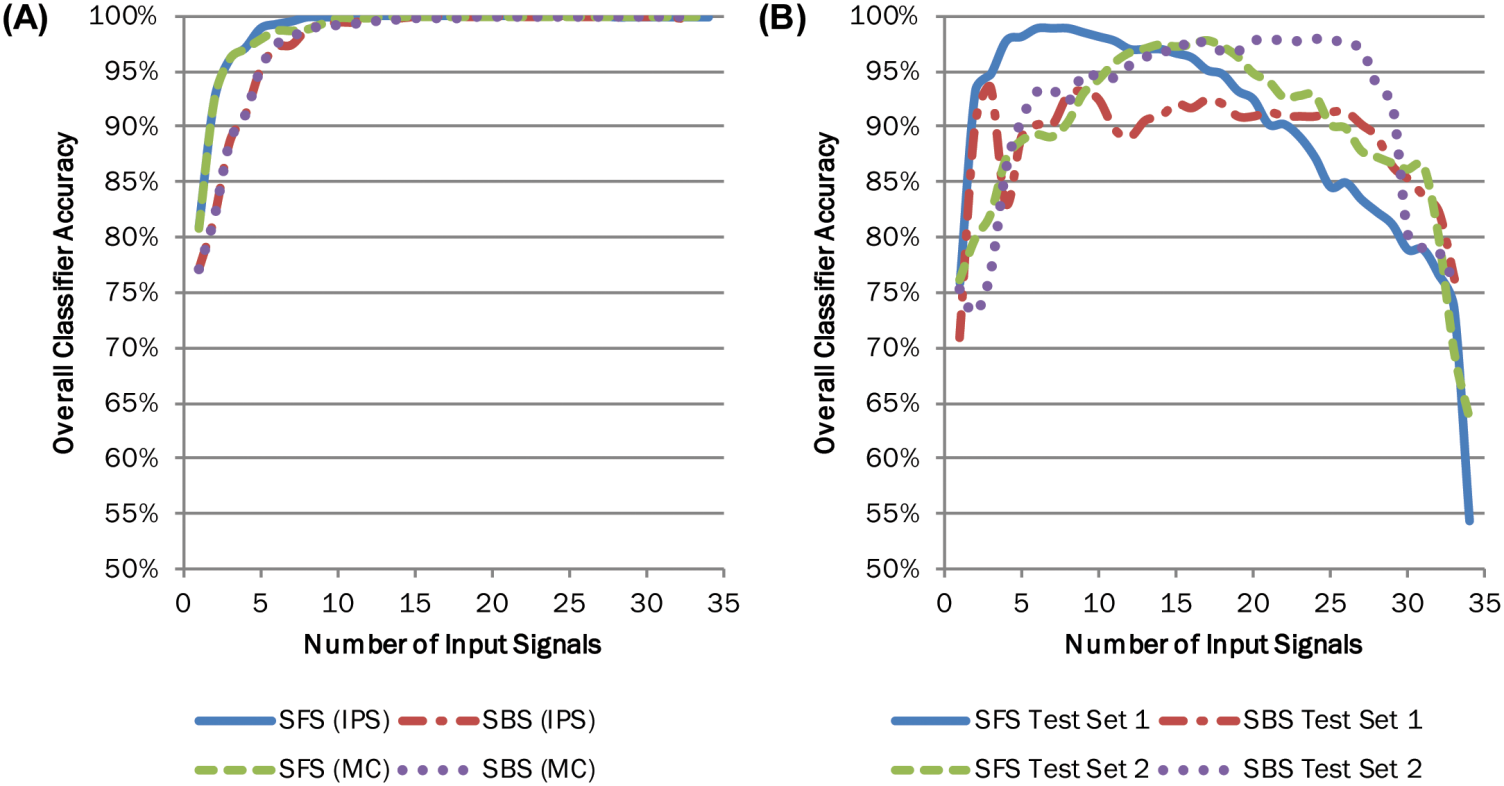
Input signal optimization led to at least 90% classifier accuracy regardless of evaluation dataset. Classifiers were trained on the full set of trial input signals from three subjects walking with their clinically prescribed ankle-foot prosthesis when they could see the configuration of the cross-slope. Input signals were added to the classifiers using sequential forward selection (SFS) and sequential backward selection (SBS) based on accuracy classifying the evaluation dataset. (A) Classifiers were evaluated using data from the training set via leave-one-out cross-validation with (IPS) and without (MC) in-pylon sensor data. (B) Classifiers were also evaluated using data from a subject walking with his clinically prescribed ankle-foot prosthesis when he could not see the configuration of the cross-slope (Test Set 1) or from two subjects walking with the prototype ankle-foot prosthesis when they could see the configuration of the cross-slope (Test Set 2) with in-pylon sensor data.

The effect of adding input signals to the classifier differed when accuracy was evaluated using LOOCV and when other test data was used. For LOOCV, accuracy increased to 100% and was maintained when additional input signals were added (Fig 3A). SFS did not require as many input signals as SBS to reach 100% accuracy. When evaluated using test data, there was a peak in classifier accuracy, after which using additional input signals decreased the accuracy (Fig 3B). More input signals were required to accurately predict the cross-slope encountered in Test Set 2 than Test Set 1 (Fig 3B). However, similar levels of accuracy could be achieved. For both test datasets, accuracy was not as high as that found using LOOCV.

**Fig 4:**
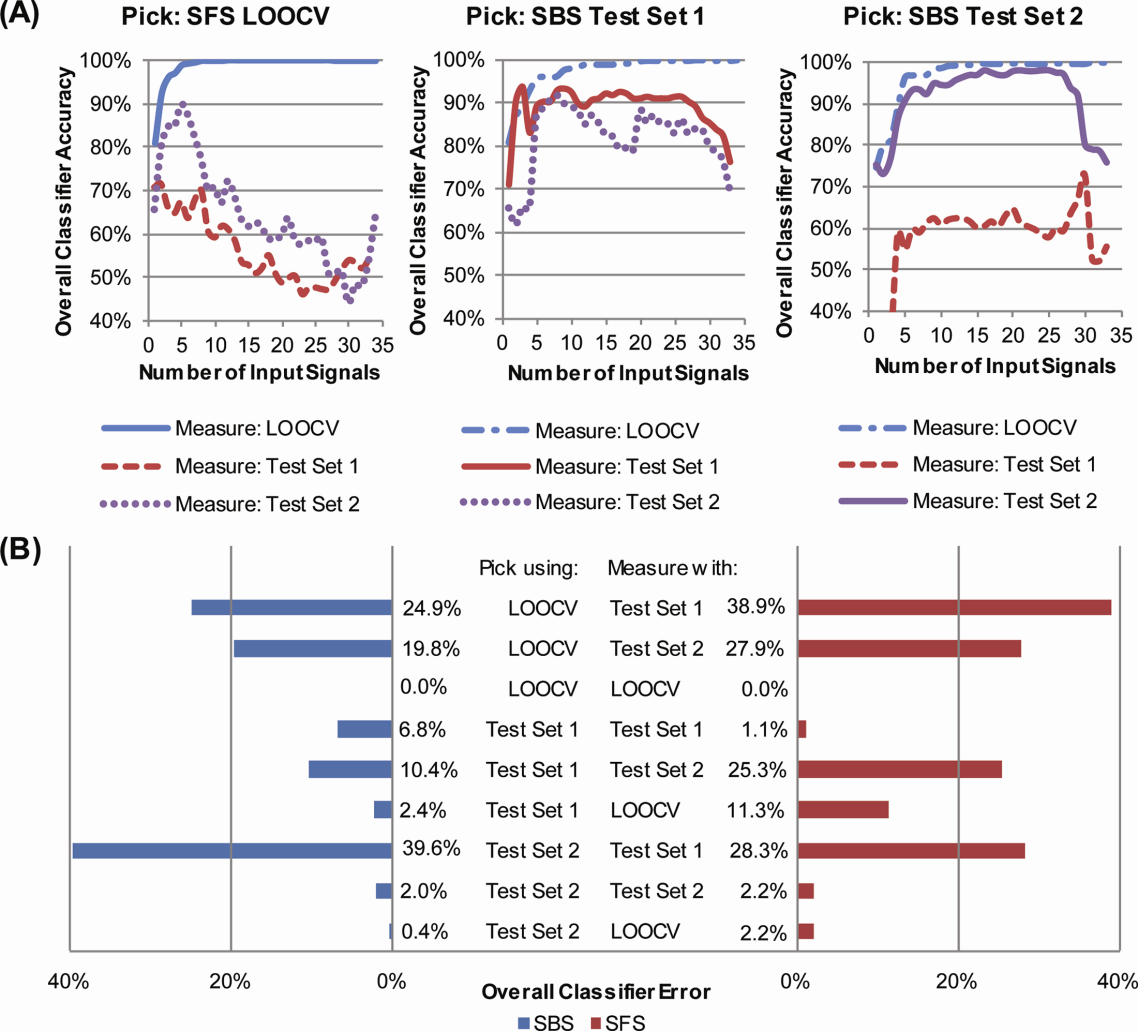
Classifier accuracy varies when evaluated using different datasets but can be optimized for versatility. All classifiers were trained using data from three subjects walking with their clinically prescribed ankle-foot prosthesis when they could see the configuration of the cross-slope. All test and training datasets included in-pylon sensor data. (A) A series of classifiers constructed using sequential forward selection (SFS) or sequential backward selection (SBS) to add input signals to the classifiers (Pick) based on overall classifier accuracy determined using leave-one-out cross-validation with the training dataset (LOOCV, left), data from a subject walking with his clinically prescribed ankle-foot prosthesis when he could not see the configuration of the cross-slope (Test Set 1, middle) and data from two subjects walking with the prototype ankle-foot prosthesis when they could see the configuration of the cross-slope (Test Set 2, right) were evaluated (Measure) using LOOCV (blue), Test Set 1 (red) and Test Set 2 (purple). The classifier accuracy shown with solid lines was also used to determine the order in which input signals were added. (B) Overall classifier error for the most accurate classifiers found using SFS and SBS to pick input signals based on overall accuracy determined using LOOCV, Test Set 1 and Test Set 2. Error was measured by evaluating all six classifiers using data from Test Set 1, data from Test Set 2 and LOOCV.

Using the full set of trial input signals, classifier accuracy when evaluated using LOOCV could be greater than 99% (Fig 3; Table 5). However, when these classifiers were evaluated with Test Sets 1 and 2, classifier accuracy degraded (Table 5; Fig 4). In all but one case, classification accuracy of at least one of the individual terrains was 90% or higher when the classifier was evaluated using Test Set 1 or 2 (S3 Table). For the classifiers with the least amount of input signals that reached greater than 99% accuracy measured using LOOCV, those with input signals chosen using SFS had slightly better accuracy when evaluated using Test Set 1 or 2 than those with input signals chosen using SBS (Table 5). However, the opposite was true for the most accurate classifiers found using overall accuracy measured using LOOCV (i.e., the classifier that reached 100% measured accuracy with the least amount of input signals, n = 12 for SFS and n = 15 for SBS), Test Set 1 (n = 6 for SFS, n = 9 for SBS) and Test Set 2 (n = 17 for SFS, n = 16 for SBS) (Fig 4B). When measured with a different dataset than the one used to choose input signals, classifier accuracy did not necessarily respond in the same manner as the number of input signals increased and was non-monotonic when not evaluated with LOOCV (Fig 4A). Error was lowest when accuracy was measured using the dataset for which input signals were picked with SFS or when evaluated using LOOCV. When input signals included in-pylon sensor data and were chosen using LOOCV, error was lower when measured using Test Set 2 than that measured using Test Set 1 (Fig 4B; Table 5).

**Table 5:** Overall accuracy (All) and range of accuracy for individual cross-slope terrain (Ind.) for the classifier with the fewest input signals that correctly identified at least 99% of the cross-slope terrain using sequential forward selection (SFS) and sequential backward selection (SBS).

	Input Signals			Accuracy		
				LOOCV Mid-Swing	Test Set 1 Mid-Swing	Test Set 2 Mid-Swing
SFS (IPS)	1 Ankle Inversion α 2 Shank Vert Velocity 3 Ankle Flexion	4 IPS AP Acceleration 5 Foot ML Velocity 6 Foot Vert Velocity	All	0.99	0.64	0.87
			Ind.	0.98–1.00	0.41–0.90	0.84–0.94
SFS (MC)	1 Ankle Inversion α 2 Shank Vert Velocity 3 Ankle Flexion 4 Ankle Flexion α 5 Shank Vert AngVel*	6 Shank Vert Acc* 7 Ankle Inversion ω 8 Shank ML Velocity 9 Shank ML Acc*	All	0.99	0.88	0.83
			Ind.	0.99–1.00	0.74–1.00	0.54–1.00
SBS (IPS)	1 Ankle Inversion ω 2 Foot Vert Acc 3 Foot ML AngVel 4 Foot AP AngVel 5 Shank AP Velocity	6 Ankle Flexion 7 ML COP 8 Foot Vert AngVel 9 Foot ML Acc	All	0.99	0.62	0.76
			Ind.	0.98–1.00	0.19–1.00	0.63–1.00
SBS (MC)	1 Ankle Inversion ω 2 Foot Vert Acc 3 Foot ML AngVel 4 Foot AP AngVel 5 Shank AP Velocity	6 Foot ML Acc 7 Shank Vert AngVel* 8 ML COP 9 AP COP	All	0.99	0.69	0.46
			Ind.	0.99–1.00	0.35–1.00	0.23–0.83

LOOCV, classifier accuracy evaluated using leave-one-out cross-validation with the training data from three subjects walking with their clinically prescribed ankle-foot prosthesis when they could see the configuration of the cross-slope; Test Set 1, classifier accuracy evaluated using data from a subject walking with his clinically prescribed ankle-foot prosthesis when he could not see the configuration of the cross-slope; Test Set 2, classifier accuracy evaluated using data from two subjects walking with the prototype ankle-foot prosthesis when they could see the configuration of the cross-slope; IPS, in-pylon sensor data used for shank angular velocity and acceleration measurements; MC, motion capture data used for shank angular velocity and acceleration measurements;

* indicates an input signal calculated using motion capture data was used for an analogous signal measured using in-pylon sensors; Definitions: ω, angular velocity; α, angular acceleration; COP, center of pressure; ML, mediolateral direction; AP, anteroposterior direction; Vert, vertical direction; AngVel, angular velocity; Acc, acceleration.

## Discussion

We explored terrain prediction in a novel application, a cross-slope exhibiting uneven terrain in the mediolateral direction, to determine if a relatively simple feature set and classifier could accurately predict the cross-slope terrain when subjects could and could not see (and adjust to) the surface prior to stepping on it. LDA classifiers were trained and their accuracy was evaluated with various combinations of kinematic and kinetic signals measured during mid-swing as transtibial amputees traversed level ground and cross-slopes. While in-pylon sensor data alone was insufficient to accurately predict the type of cross-slope encountered, including additional input signals, especially foot and ankle kinematics, led to classifiers with greater than 90% accuracy. Foot-mounted sensors have been used to measure prosthetic ankle angle [12] and foot kinematics can be measured using an accelerometer and gyroscope, similar to the pylon-mounted sensors used in the experiment. If sensors are mounted on the prosthetic foot in addition to the pylon, the present results suggest a simple, real-time classifier can be used to predict cross-slope terrains for lower-limb amputees.

As expected, the classifier needed similar input signals to achieve high accuracy regardless of whether in-pylon sensor data or motion capture data supplied the acceleration and angular velocity of the residual shank. This may be due in part to the high importance of some signals for which only motion capture data were available, such as ankle inversion angular velocity. However, similar input signal priority with and without in-pylon sensor data is consistent with previous work, which has shown that measurements of leg kinematics from body-worn sensors are comparable to those gathered using motion capture [28] and that classifiers based on accelerometer and motion capture data can determine the cause of a fall with equal accuracy [29]. When accuracy was evaluated using LOOCV, the same input signals were selected for classifiers that had 95% overall accuracy. In addition, the number of signals added to reach 99% accuracy was similar, although not the same group of signals. For SFS, this difference stems from selection of an in-pylon sensor signal but not the associated motion capture signal. Similarly, for SBS, when removing signals, classifier accuracy was not quite the same using motion capture values compared to that when using in-pylon sensor measurements, so signals were removed in a different order. Interestingly, of the 15 signals selected by SBS (9) and SFS (6) to reach 99% accuracy with in-pylon sensor data available, only 1 in-pylon sensor measurement was selected while 4 of the 18 signals selected to reach 99% accuracy were motion capture data substitutions for the in-pylon sensor measurements. This may be a result of the difference in reference frame between the motion-capture-measured shank acceleration and angular velocity, which were measured in a global frame, while the in-pylon-sensor-measured values were measured in a shank reference frame. Considering the similarity in performance and input signal priority for classifiers using in-pylon or motion capture data, well-tuned prosthesis-mounted sensor data can likely be substituted for motion-capture data for prediction of cross-slope terrain.

Examination across all three test datasets and the two training methods revealed that all of the potential input signals except for ankle flexion moment were added to form the best classifier at least once. Foot-floor interaction (i.e., COPs and GRFs), moment and power signals were not as important to classifier accuracy as ankle angle, angular velocity and angular acceleration or segment velocity, acceleration and angular velocity. Inversion angular velocity and acceleration are more critical than flexion angular velocity or acceleration, but flexion angle is used more often than inversion angle. Inversion angular velocity and acceleration are important in predicting cross-slope terrain as they indicate the change in angle caused by the cross-slope and the rate at which that change is occurring. A cross-slope induces larger changes in inversion angle than in flexion angle during stance, but there are adaptations in both angles. Foot and shank velocity are both important for classifier accuracy but foot acceleration and angular velocity are more often used than those of the shank. Changes in foot acceleration and, to a lesser extent, angular velocity caused by the cross-slope are more distinctive than those of the shank and thus may provide a wider difference between the terrain types that is more readily recognized by the classifier.

As expected, evaluation using LOOCV with the training dataset led to higher accuracy measures than evaluation using one of the test datasets, when subjects could not anticipate the cross-slope terrain (Test Set 1) or walked with the prototype prosthesis (Test Set 2). However, contrary to expectations, the input signals selected based on accuracy results when classifiers were evaluated with LOOCV were only moderately similar to those selected based on accuracy results when classifiers were evaluated with test data. Only one input signal, inversion angular velocity, was used in five of the six classifiers and one other, foot vertical velocity, in four. Nine more input signals were used in three classifiers. Accuracy when evaluated with the same test set as that used to select the input signals was comparable to that found in literature for locomotion mode classifier accuracy during steady state locomotion (e.g., [13, 16, 20]). When evaluated using a different test set than the one used to select input signals, accuracy was comparable to locomotion mode classifier accuracy during transitions between modes [15].

When evaluated using the test datasets, including additional input signals did not always improve accuracy. This is likely due to overfitting of the data. If the number of training samples is limited, classifier error tends to reach a minimum and then increase as more input signals are added [30, 31]. With a fixed set of training data, the classifier can be trained to perfectly discriminate among cross-slopes in the training set, but such a classifier will not generalize well to new datasets. Evaluating accuracy on other datasets is one way to ensure that the classifier can be generalized to new subjects or new conditions [32]. Choosing input signals based on the accuracy measurements when evaluated with Test Set 1, when the subjects could not anticipate the cross-slope terrain, produced the classifier that was most accurate across all of the test datasets. For this classifier, the worst error was less than 11%, which is toward the upper range of some of the steady state errors found for locomotion mode classifiers in literature (e.g., [13, 15, 16]). While many locomotion mode classifiers are trained specifically for individual subjects or unblinded conditions, the ability of the cross-slope classifier to reach similar accuracy when trained and evaluated on data from different subjects and conditions (e.g., visible vs. obscured cross-slope, clinically prescribed vs. prototype ankle-foot prosthesis) suggests that it may be easily implemented whether the amputee can anticipate the cross-slope terrain or not. Consider the real-world example of walking on a lumpy grass lawn at night with a controller trained in daylight. The person cannot see the terrain at night, but they know it might be uneven. Our results show the algorithm could work under these conditions. Other conditions (e.g., clinically prescribed vs. prototype ankle-foot prosthesis), suggest the algorithm could also work when switching to a different prosthesis.

Different input signal selection methods were used primarily to gauge variation in signals selected and the impact of different input signal groups on classifier accuracy. While previous work classifying a walking task in transfemoral amputees found that SBS and SFS selected similar input signals (mostly surface electromyography measurements) and thus produced similar classifier accuracy [14], other work has shown that the performance of feature selection algorithms can change with different training datasets or applications [33]. When only in-pylon sensor data were used, there was little difference in signal selection or classifier accuracy. When the full set of input signals were used, SBS typically required more signals to achieve similar classifier accuracy as SFS and up to one-third of the signals selected were chosen by both algorithms. Increasing the number of possible input signals from just in-pylon sensor data to the full set increased variation in signal selection. The small subset of signals selected by both SBS and SFS strongly influence classifier accuracy as they were selected in both cases despite different decision-making algorithms. Though similar accuracy can be achieved with different groups of input signals, using the set of input signals identified by SFS would require fewer sensors.

While a set of useful input signals were identified for predicting a cross-slope, this study has some potential limitations. Although a set of input signals useful for predicting the cross-slope with the prototype ankle-foot prosthesis (Test Set 2) was found, the prototype prosthesis was not allowed to adapt to the cross-slope in the dataset that was tested. Thus, one potential limitation is that patterns will shift and input signal contribution to classifier accuracy may change when the prototype prosthesis can adapt. However, signals were chosen based on the mid-swing classifier, which was trained and evaluated using windows of data from mid-swing up to 75 ms after heel-strike. In a real-time control scheme, the classifier will continuously examine windows of data to determine the cross-slope then update prosthesis configuration for the step using a majority voting scheme that considers cross-slope determinations from a set of the most recent data windows, similar to previous approaches (e.g., [14, 15, 18]). The number of windows included in the voting scheme will be determined experimentally, but the cross-slope determination will be made by 75 ms after heel strike and set for the duration of the step. Once the cross-slope is determined, the semi-active prototype prosthesis will adjust stiffness accordingly. While a small processing delay for cross-slope determination may exist depending on the exact hardware implementation, the simplicity of the LDA classifier combined with continuous classification based on a voting scheme, careful hardware selection, and optimization of code for efficiency should render any delay imperceptible to the amputee [18]. Previous work with prosthesis locomotion mode switching (e.g., level-ground, stairs or slopes) has shown that classifiers, both similar to and more complex than those presented here, designed offline can be successfully implemented in real-time and exhibit accuracy that is correlated, if not similar, to offline accuracy (e.g., [14, 34]). As the prototype prosthesis adaptation will not occur until after heel-strike plus a short lag period and acts the same during mid-swing whether or not adaptation is enabled, the patterns for the mid-swing classifier are unlikely to change significantly enough to affect which input signals are most useful for classifier accuracy. These results are a first step toward determining classifier structure, which may need adjustment after implementation with the semi-active device when the effects of adaptation and misclassification on classifier performance in subsequent steps can be examined. Another potential limitation is that input signals were selected based on data from a specific set of subjects and may not be as effective with new subjects. Using test data from the original subjects in conditions modified from those of the training data broadened the set of input signals identified as useful and mitigates the risk of excluding input signals critical to classifier accuracy. Since these LDA classifiers are relatively quick to train (less than five minutes), classifier performance on a new subject could be improved by adding a few trials from the new subject to the training set and retraining before full implementation. This approach has successfully improved locomotion mode classification with a minimal amount of additional training data collected from the new subject [23].

Future work will seek to assess the classifier performance in an experimental case study examining the ability of the classifiers to predict cross-slopes encountered in real-time and using prosthesis-mounted sensors for all measurements instead of motion capture. The classifiers will also be coupled with the prototype variable-stiffness prosthesis and used to select a stiffness profile designed to allow the foot to conform to the cross-slope. When implemented with the prototype variable-stiffness prosthesis, the classifiers will likely classify a range of slopes smaller and larger than the 15° slopes on which it was trained as inverting or everting surfaces. Since the prototype prosthesis adjusts stiffness in response to a detected cross-slope, it will adjust the stiffness based on the type of slope detected and conform to the actual surface. Thus, the actual resolution of the prosthesis will be finer than 15°. Future work should investigate the range of cross-slopes classified as flat, inverting, and everting as well as amputee responses to adaptation, the functional consequences of a misclassification, and if the prosthesis adaptation should be tuned to smaller variations in cross-slopes. Future work should also explore other classifier types, such as support vector machines, and other training algorithms to improve classifier performance and generalizability to new subjects. Finally, the extension of the classifier to predict other walking conditions where coronal-plane adaptation may be beneficial, such as turning, should be investigated.

In summary, this work has shown measurements of residual limb kinematics can be used by an LDA classifier to successfully predict a cross-slope with high accuracy. While in-pylon sensors alone are insufficient, addition of foot and ankle kinematic sensors on the prosthetic foot may provide enough additional information to accurately predict cross-slope terrain.

## Supporting information

S1 Table List of input signal sets available to the classifier. Input signals included ankle, foot and shank kinematic, ankle moment and power, ground reaction force (GRF), center of pressure (COP) and in-pylon sensor (IPS) data.
(DOCX)

S2 Table Confusion matrices (CFM) for classifiers with 2 to 6 input signals that exhibited the highest overall accuracy when only information from the in-pylon sensors was used. (DOCX)

S3 Table Confusion matrices (CFM) for the classifiers found using sequential forward selection (SFS) and sequential backward selection (SBS) that, with the fewest input signals, correctly identified at least 99% of the cross-slopes. (DOCX)

S1 Dataset Test Set 1. Possible input signals collected from a transtibial amputee walking with his clinically prescribed ankle-foot prosthesis when he could not see the cross-slope (eversion, flush or inversion).
(TXT)

S2 Dataset Test Set 2. Possible input signals collected from two transtibial amputees walking with the new prototype prosthesis with adaptation disabled when they could see the cross-slope (eversion, flush or inversion).
(TXT)

S3 Dataset Training data. Possible input signals collected from three transtibial amputees walking with their clinically prescribed ankle-foot prosthesis when they could see the cross-slope (eversion, flush or inversion).
(TXT)
