# Supplementary material for: Identifying classifier input signals to predict a cross-slope during transtibial amputee walking
Source: PLoS One. 2018 Feb 16;13(2):e0192950. doi: 10.1371/journal.pone.0192950 (PMC5815617; doi:10.1371/journal.pone.0192950)
Supplement: S1 Table — Input signals included ankle, foot and shank kinematic, ankle moment and power, ground reaction force (GRF), center of pressure (COP) and in-pylon sensor (IPS) data. (DOCX) [file pone.0192950.s001.docx]

**S1 Table. List of input signal sets available to the classifier.** Input signals included ankle, foot and shank kinematic, ankle moment and power, ground reaction force (GRF), center of pressure (COP) and in-pylon sensor (IPS) data.

| **Full Set of Trial Input Signals** | 1. Ankle Flexion 2. Ankle Inversion 3. Ankle Flexion Angular Velocity 4. Ankle Inversion Angular Velocity 5. Ankle Flexion Angular Acceleration 6. Ankle Inversion Angular Acceleration 7. Ankle Flexion Moment 8. Ankle Inversion Moment 9. Ankle Flexion Power 10. Ankle Inversion Power 11. Shank Mediolateral Velocity 12. Shank Anteroposterior Velocity | 1. Shank Vertical Velocity 2. Foot Mediolateral Velocity 3. Foot Anteroposterior Velocity 4. Foot Vertical Velocity 5. IPS/Shank Mediolateral Acceleration 6. IPS/Shank Anteroposterior Acceleration 7. IPS/Shank Vertical Acceleration 8. Foot Mediolateral Acceleration 9. Foot Anteroposterior Acceleration 10. Foot Vertical Acceleration | 1. IPS Coronal-Plane/Shank Anteroposterior Angular Velocity 2. IPS Sagittal-Plane/Shank Mediolateral Angular Velocity 3. IPS Transverse-Plane/Shank Vertical Angular Velocity 4. Foot Anteroposterior Angular Velocity 5. Foot Mediolateral Angular Velocity 6. Foot Vertical Angular Velocity 7. Mediolateral GRF 8. Anteroposterior GRF 9. Vertical GRF 10. Mediolateral COP 11. Anteroposterior COP 12. Vertical COP |
| --- | --- | --- | --- |
| **In-Pylon Sensor Input Signals** | 1. IPS Mediolateral Acceleration 2. IPS Anteroposterior Acceleration | 1. IPS Vertical Acceleration 2. IPS Coronal-Plane Angular Velocity | 1. IPS Sagittal-Plane Angular Velocity 2. IPS Transverse-Plane Angular Velocity |
