# Supplementary material for: Identifying classifier input signals to predict a cross-slope during transtibial amputee walking
Source: PLoS One. 2018 Feb 16;13(2):e0192950. doi: 10.1371/journal.pone.0192950 (PMC5815617; doi:10.1371/journal.pone.0192950)
Supplement: S2 Table — (DOCX) [file pone.0192950.s002.docx]

**S2 Table.** **Confusion matrices (CFM) for classifiers with 2 to 6 input signals that exhibited the highest overall accuracy when only information from the in-pylon sensors was used.**

| **Input Signals** | |  | **LOOCV**  **Mid-Swing CFM** | | | **Test Set 1**  **Mid-Swing CFM** | | | **Test Set 2**  **Mid-Swing CFM** | | |
| --- | --- | --- | --- | --- | --- | --- | --- | --- | --- | --- | --- |
|  |  |  | Ev | Fl | Inv | Ev | Fl | Inv | Ev | Fl | Inv |
| IPS ML Acc | IPS Cor AngVel | Ev | 0.64 | 0.23 | 0.13 | 0.06 | 0.33 | 0.61 | 0.57 | 0.40 | 0.03 |
|  |  | Fl | 0.23 | 0.59 | 0.18 | 0.19 | 0.39 | 0.42 | 0.16 | 0.73 | 0.11 |
|  |  | Inv | 0.27 | 0.06 | 0.67 | 0.22 | 0.29 | 0.49 | 0.45 | 0.27 | 0.28 |
| IPS InfSup Acc  IPS ML Acc | IPS Cor AngVel | Ev | 0.71 | 0.16 | 0.13 | 0.29 | 0.15 | 0.56 | 0.88 | 0.08 | 0.04 |
|  |  | Fl | 0.20 | 0.68 | 0.12 | 0.33 | 0.23 | 0.44 | 0.44 | 0.42 | 0.14 |
|  |  | Inv | 0.19 | 0.14 | 0.67 | 0.40 | 0.06 | 0.54 | 0.63 | 0.07 | 0.30 |
| IPS AP Acc  IPS InfSup Acc  IPS ML Acc | IPS Cor AngVel | Ev | 0.69 | 0.15 | 0.16 | 0.21 | 0.33 | 0.46 | 0.74 | 0.26 | 0.00 |
|  |  | Fl | 0.15 | 0.79 | 0.06 | 0.27 | 0.43 | 0.30 | 0.29 | 0.67 | 0.04 |
|  |  | Inv | 0.18 | 0.10 | 0.72 | 0.42 | 0.15 | 0.42 | 0.58 | 0.30 | 0.12 |
| IPS AP Acc  IPS InfSup Acc  IPS ML Acc | IPS Cor AngVel  IPS Sag AngVel | Ev | 0.73 | 0.12 | 0.15 | 0.30 | 0.18 | 0.51 | 0.77 | 0.20 | 0.03 |
|  |  | Fl | 0.12 | 0.81 | 0.07 | 0.16 | 0.56 | 0.28 | 0.39 | 0.53 | 0.08 |
|  |  | Inv | 0.20 | 0.06 | 0.73 | 0.41 | 0.14 | 0.44 | 0.58 | 0.22 | 0.20 |
| IPS AP Acc  IPS InfSup Acc  IPS ML Acc | IPS Cor AngVel  IPS Tran AngVel  IPS Sag AngVel | Ev | 0.71 | 0.13 | 0.16 | 0.34 | 0.21 | 0.45 | 0.61 | 0.33 | 0.07 |
|  |  | Fl | 0.11 | 0.87 | 0.02 | 0.24 | 0.52 | 0.24 | 0.50 | 0.47 | 0.02 |
|  |  | Inv | 0.17 | 0.07 | 0.76 | 0.54 | 0.20 | 0.26 | 0.39 | 0.25 | 0.36 |

LOOCV, classifier accuracy evaluated using leave-one-out cross-validation with the training data from three subjects walking with their clinically prescribed ankle-foot prosthesis when they could see the configuration of the cross-slope; Test Set 1, classifier accuracy evaluated using data from a subject walking with his clinically prescribed ankle-foot prosthesis when he could not see the configuration of the cross-slope; Test Set 2, classifier accuracy evaluated using data from two subjects walking with the prototype ankle-foot prosthesis when they could see the configuration of the cross-slope; Ev, eversion; Fl, flush; Inv, inversion; Definitions: AngVel, residual limb shank angular velocity; Acc, residual limb shank acceleration; Cor, coronal plane; Tran, transverse plane; Sag, sagittal plane; AP, anteroposterior direction; InfSup, inferior-superior direction; ML, mediolateral direction. The diagonal entries in the confusion matrices, which represent classifier accuracy for individual cross-slope terrains, are colored red if less than 60% and yellow if greater than 60% but less than 90%.
