# Supplementary material for: Identifying classifier input signals to predict a cross-slope during transtibial amputee walking
Source: PLoS One. 2018 Feb 16;13(2):e0192950. doi: 10.1371/journal.pone.0192950 (PMC5815617; doi:10.1371/journal.pone.0192950)
Supplement: S3 Table — (DOCX) [file pone.0192950.s003.docx]

**S3 Table. Confusion matrices (CFM) for the classifiers found using sequential forward selection (SFS) and sequential backward selection (SBS) that, with the fewest input signals, correctly identified at least 99% of the cross-slopes.**

|  | **Input Signals** | |  | **LOOCV Mid-Swing CFM** | | | | | **Test Set 1 Mid-Swing CFM** | | | **Test Set 2 Mid-Swing CFM** | | |
| --- | --- | --- | --- | --- | --- | --- | --- | --- | --- | --- | --- | --- | --- | --- |
|  |  |  |  | Ev | Fl | | Inv | | Ev | Fl | Inv | Ev | Fl | Inv |
| SFS (IPS) | 1. Ankle Inversion α 2. Shank Vert Velocity 3. Ankle Flexion | 1. IPS AP Acc 2. Foot ML Velocity 3. Foot Vert Velocity | Ev | 0.98 | | 0.01 | | 0.01 | 0.90 | 0.10 | 0.00 | 0.84 | 0.00 | 0.16 |
|  |  |  | Fl | 0.00 | | 1.00 | | 0.00 | 0.29 | 0.66 | 0.05 | 0.15 | 0.85 | 0.00 |
|  |  |  | Inv | 0.00 | | 0.00 | | 1.00 | 0.21 | 0.38 | 0.41 | 0.06 | 0.00 | 0.94 |
| SFS (MC) | 1. Ankle Inversion α 2. Shank Vert Velocity 3. Ankle Flexion 4. Ankle Flexion α 5. Shank Vert AngVel* | 1. Shank Vert Acc* 2. Ankle Inversion ω 3. Shank ML Velocity 4. Shank ML Acc* | Ev | 0.99 | | 0.00 | | 0.01 | 0.93 | 0.07 | 0.00 | 0.54 | 0.13 | 0.33 |
|  |  |  | Fl | 0.01 | | 0.99 | | 0.00 | 0.00 | 1.00 | 0.00 | 0.00 | 1.00 | 0.00 |
|  |  |  | Inv | 0.00 | | 0.00 | | 1.00 | 0.03 | 0.23 | 0.74 | 0.07 | 0.01 | 0.92 |
| SBS (IPS) | 1. Ankle Inversion ω 2. Foot Vert Acc 3. Foot ML AngVel 4. Foot AP AngVel 5. Shank AP Velocity | 1. Ankle Flexion 2. ML COP 3. Foot Vert AngVel 4. Foot ML Acc | Ev | 0.98 | | 0.00 | | 0.02 | 0.80 | 0.20 | 0.00 | 0.63 | 0.02 | 0.35 |
|  |  |  | Fl | 0.00 | | 1.00 | | 0.00 | 0.00 | 1.00 | 0.00 | 0.00 | 0.68 | 0.32 |
|  |  |  | Inv | 0.00 | | 0.00 | | 1.00 | 0.11 | 0.70 | 0.19 | 0.00 | 0.00 | 1.00 |
| SBS (MC) | 1. Ankle Inversion ω 2. Foot Vert Acc 3. Foot ML AngVel 4. Foot AP AngVel 5. Shank AP Velocity | 1. Foot ML Acc 2. Shank Vert AngVel* 3. ML COP 4. AP COP | Ev | 0.99 | | 0.00 | | 0.01 | 0.84 | 0.16 | 0.00 | 0.27 | 0.73 | 0.00 |
|  |  |  | Fl | 0.01 | | 0.99 | | 0.00 | 0.00 | 1.00 | 0.00 | 0.02 | 0.83 | 0.15 |
|  |  |  | Inv | 0.00 | | 0.00 | | 1.00 | 0.00 | 0.65 | 0.35 | 0.00 | 0.77 | 0.23 |

LOOCV, classifier accuracy evaluated using leave-one-out cross-validation with the training data from three subjects walking with their clinically prescribed ankle-foot prosthesis when they could see the configuration of the cross-slope; Test Set 1, classifier accuracy evaluated using data from a subject walking with his clinically prescribed ankle-foot prosthesis when he could not see the configuration of the cross-slope; Test Set 2, classifier accuracy evaluated using data from two subjects walking with the prototype ankle-foot prosthesis when they could see the configuration of the cross-slope; Ev, eversion; Fl, flush; Inv, inversion; IPS, in-pylon sensor data used for shank angular velocity and acceleration measurements; MC, motion capture data used for shank angular velocity and acceleration measurements; * indicates an input signal calculated using motion capture data was used for an analogous signal measured using in-pylon sensors; Definitions: ω, angular velocity; α, angular acceleration; COP, center of pressure; ML, mediolateral direction; AP, anteroposterior direction; Vert, vertical direction; AngVel, angular velocity; Acc, acceleration. The diagonal entries in the confusion matrices, which represent classifier accuracy for individual cross-slope terrains, are colored red if less than 60%, yellow if between 60% and 90% and green if greater than 90%.
